# Wave intensity, an index of ventriculo-arterial interaction, increases in hypertensive subjects but decreases in normotensive subjects during the cold pressor test

**DOI:** 10.1007/s10396-020-01077-9

**Published:** 2021-01-16

**Authors:** Midori Tanaka, Motoaki Sugawara, Kiyomi Niki, Yasuo Ogasawara

**Affiliations:** 1 Faculty of Health Care Sciences, Himeji Dokkyo University, 7-2-1 Kamiohno, Himeji, Hyogo Japan; 2 Faculty of Medicine, Tokyo Women’s Medical University, Tokyo, Japan; 3 Department of Cardiology, Niki Heart Clinic, Tokyo, Japan; 4 Department of Medical Engineering, Kawasaki Medical School, Kurashiki, Japan

**Keywords:** Cardiac contractile index, Wave intensity, Carotid echo, Hypertension

## Abstract

**Purpose:**

Cardiovascular reactivity to the cold pressor test (CPT) is considered to be a marker for apparent and potential hypertension. We aimed to elucidate the association between the changes in wave intensity (WI) during CPT and hypertension.

**Methods:**

We recruited 85 volunteers, 33 of whom were hypertensive and 52 normotensive. Using ultrasonic equipment during CPT, we measured carotid arterial WI, which is defined in terms of blood pressure and velocity in the carotid artery.

**Results:**

The peak WI (W_1_) increased during CPT in 70.6% of hypertensive individuals, but decreased in 72.6% of normotensive individuals. The chi-square (χ^2^) test showed that the association between the direction of change in W_1_ (increase or decrease) and the blood pressure (hypertensive or normotensive) was very strong (*P* < 0.0001).

**Conclusion:**

Direction of change in W_1_ during CPT is a clear marker to discriminate cardiovascular reactivity that does not vary depending on each investigator’s subjective point of view.

## Introduction

Hypertensive persons are considered to be hyper-reactive to CPT. A widely accepted indicator for reactivity is the amount of increase in systolic blood pressure [1, 2]. However, the threshold value to discriminate between hyper-reactive and normo-reactive subjects varies with the investigator. Wave intensity (WI) is a hemodynamic index that can be used to evaluate the working condition of the heart interacting with the arterial system. It can be defined at any site in the circulatory system in terms of blood velocity and blood pressure, and provides integrated information [3]. We suppose that WI has the ability to discriminate between hyper-reactors and normo-reactors more clearly. To evaluate this supposition, we measured WI in hypertensive and normotensive subjects during CPT.

## Materials and methods

### Subjects

Subjects who had a blood pressure lower than 130/80 mmHg were categorized as normotensive. We recruited 52 normotensive subjects (Norm) from among students and staff at Himeji Dokkyo University, Himeji, Japan. We recruited 33 hypertensive subjects (HT) from among outpatients who were diagnosed as hypertensive at Sakakibara Heart Institute, Tokyo, Japan. All subjects had no abnormal ECGs and had normal echocardiographic findings. Baseline characteristics of the subjects are shown in Table 1.

**Table 1:** Baseline characteristics of the subjects

Total (*n* = 85)	Hypertensive (*n* = 33)	Normotensive (*n* = 52)	P-value
Age (years)	65 ± 7	41 ± 16	*P* < 0.0001
Male (*n*)/Female (*n*)	20/13	35/17	*P* = 0.53
Heart rate (beats/min)	62 ± 10	65 ± 10	n.s
Systolic BP (mmHg)	134 ± 16	118 ± 15	*P* < 0.0001
diastolic BP (mmHg)	73 ± 9	69 ± 11	n.s
Peak value of WI (mmHg m s^–3^)	8868 ± 4244	9925 ± 5397	n.s

## Methods

### Wave intensity

Wave intensity (WI) is a hemodynamic index, which is defined as

$${\text{WI }} = \, \left( {{\text{dP}}/{\text{dt}}} \right) \, \left( {{\text{dU}}/{\text{dt}}} \right)$$

at any site in the circulatory system, where dP/dt and dU/dt are the derivatives of blood pressure (P) and velocity (U) with respect to time, respectively [3]. We developed a real-time measurement system for carotid arterial WI that simultaneously measured carotid arterial blood flow velocity and diameter. This system was incorporated in ultrasonic diagnostic equipment (SSD-6500; Aloka, Tokyo, Japan), which had a color Doppler system for blood flow velocity measurements and an echo-tracking subsystem for diameter change measurements. Using systolic and diastolic pressure measured with a cuff-type manometer applied to the upper arm, we calibrated the maximum and minimum values of a diameter change waveform and used it as a surrogate for a blood pressure waveform. We reported the details and reproducibility of this system elsewhere [3, 4].

### Cold pressor test (CPT)

**Fig. 1:**
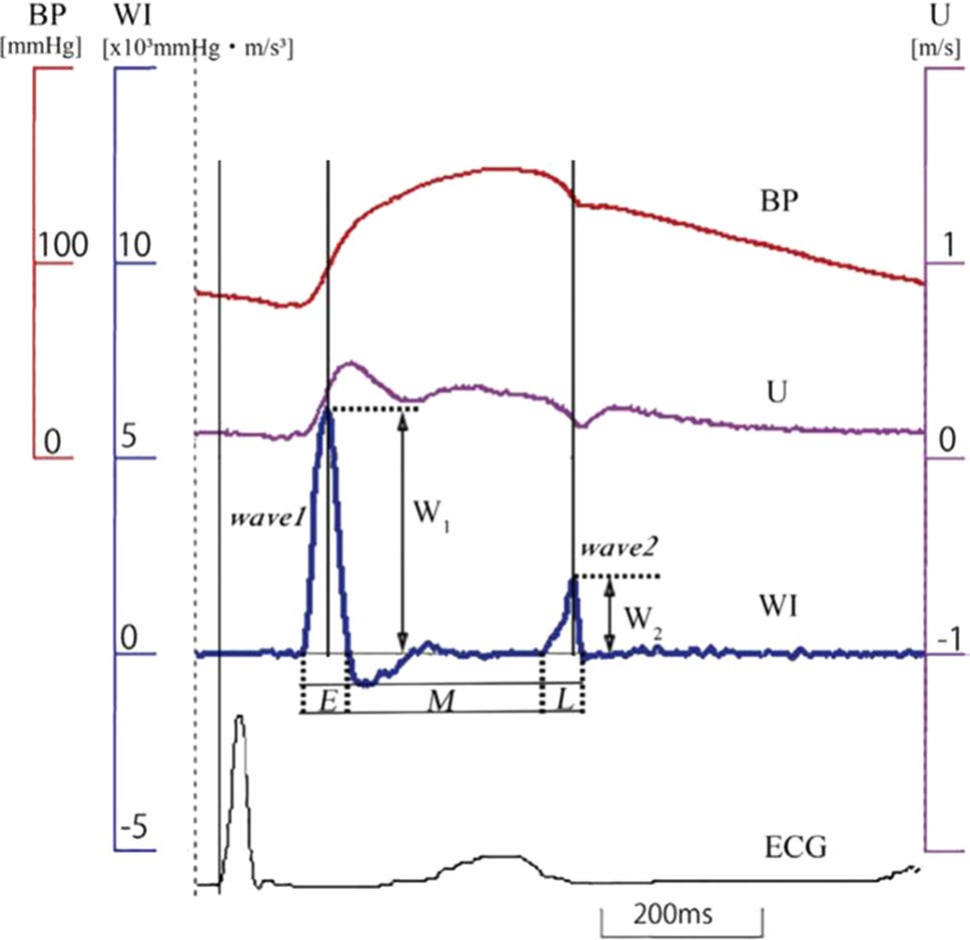
Representative recordings of blood pressure (BP), blood flow velocity (U), and calculated wave intensity (WI) in the common carotid artery, and electrocardiogram (ECG) obtained from a normal subject. WI in a normal subject shows two sharp positive peaks during a cardiac cycle, wave 1 and wave 2, and the ejection period is divided into three phases (initial (E), mid (M), and late ejection (L)) by the two waves

Each subject was asked to lie in a supine position for 10 min in a quiet room maintained at a temperature of 25℃ (resting state). Blood pressure readings were obtained during this period; the last of these measurements was used for calibrating the diameter change waveform, which is the surrogate for a blood pressure waveform. Then, the subject was asked to immerse one hand to just above the wrist for 2 min in ice water that was being kept at 0–1 °C. During the period of ice water immersion, blood pressure, blood velocity, and WI were obtained from the carotid artery, and ECG was also recorded (Fig. 1).

### Statistical analyses

Data were expressed as percent change, normalized to the index value in the resting state. Comparisons between HT and Norm groups were performed by two-way ANOVA, followed by Bonferroni test. The relationship between gender (male, female) and classification of blood pressure (HT, Norm) was analyzed by the χ^2^ test. The relationship between classification of %change in W_1_ (increase, decrease) and classification of blood pressure (HT, Norm) was also analyzed by the χ^2^ test. The relationship between %change in W_1_ (%W_1_) and age and the relationship between %W_1_ and arterial stiffness were analyzed by linear regression.

## Results

During CPT, systolic blood pressure, diastolic blood pressure, and heart rate increased significantly in both HT and Norm. Carotid arterial stroke volume did not change in either HT or Norm. Systolic carotid arterial diameter increased significantly in both HT and Norm. Diastolic carotid arterial diameter did not change in HT, but it increased significantly in Norm. The maximum velocity in the carotid artery did not change in HT, but it decreased significantly in Norm. W_1_ increased significantly in HT. On the other hand, W_1_ decreased significantly in Norm (Table 2).

**Table 2:** Influence of CPT on cardiovascular indices

Item name	Hypertensive			Normotensive		
	Rest	CPT	P-value	Rest	CPT	P-value
W_1_ (mmHg m s^–3^)	8868 ± 4244	9899 ± 4505	*p* < 0.05	9925 ± 5397	8145 ± 4797	*p* < 0.001
Systolic pressure (mmHg)	134 ± 16	158 ± 18	*p* < 0.0001	118 ± 15	135 ± 21	*p* < 0.0001
Diastolic pressure (mmHg)	73 ± 9	85 ± 11	*p* < 0.0001	69 ± 11	83 ± 14	*p* < 0.0001
Mean pressure (mmHg)	93 ± 9	109 ± 9	*p* < 0.0001	86 ± 12	100 ± 15	*p* < 0.0001
Heart rate (bpm)	62 ± 10	65 ± 11	*p* < 0.001	65 ± 10	67 ± 10	*p* < 0.01
Stroke volume (ml)	9.8 ± 2.8	9.8 ± 2.7	n.s	8.9 ± 2.3	8.9 ± 2.3	n.s
Maximum velocity (m/s)	0.47 ± 0.12	0.45 ± 0.12	n.s	0.44 ± 0.40	0.38 ± 0.34	*P* = 0.0001
Maximum diameter (mm)	8.58 ± 1.04	8.64 ± 0.97	*p* < 0.001	7.47 ± 0.92	7.63 ± 0.97	*p* < 0.01
Minimum diameter (mm)	8.21 ± 0.99	8.29 ± 0.91	n.s	7.03 ± 0.90	7.22 ± 0.93	*P* = 0.001
ß	15 ± 6	17 ± 8	n.s	9.5 ± 3.8	9.4 ± 3.5	n.s

Data are expressed as mean ± SD. *P* values were obtained by the paired *t* test

**Fig. 2:**
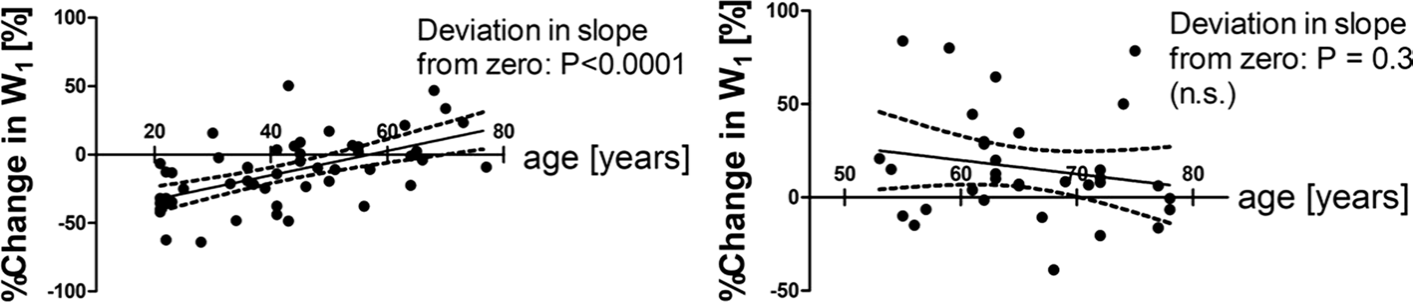
Left: Relation between the percent change in W_1_ during CPT and age in the normotensive subject group. Right: Relation between the percent change in W_1_ during CPT and age in the hypertensive subject group. Percent changes are normalized to W_1_ before CPT

Figure 2 shows the linear regression lines of %W_1_ during CPT on age. The slope of the regression line for Norm deviates significantly from zero (*r* = 0.58, *P* = 0.0001) (Fig. 2, left), while the slope of the regression line for HT does not deviate significantly from zero (*r* = – 0.19, *P* = 0.30) (Fig. 2, right).

**Fig. 3:**
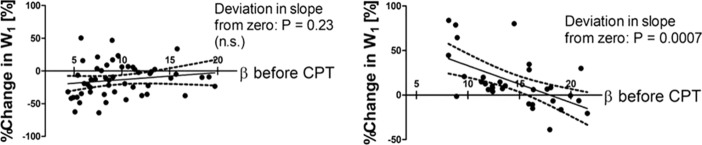
Left: Relation between the percent change in W_1_ during CPT and β before CPT (resting) in the normotensive subject group. Right: Relation between the percent change in W_1_ during CPT and β before CPT in the hypertensive subject group. Percent changes are normalized to W_1_ before CPT

To define arterial stiffness, we used the stiffness parameter β, which is considered to be independent of pressure. Figure 3 shows the linear regression lines of %W_1_ during CPT on β before CPT (resting). The slope of the regression line for Norm does not deviate significantly from zero (*r* = 0.16, *P* = 0.23), while the slope of the regression line for HT deviates significantly from zero (*r* = 0.57, P = 0.0007).

Table 3 is the contingency table in which the subjects were classified with respect to the direction of changes in W_1_ (increase or decrease) and with respect to blood pressure (HT or Norm). The χ^2^ test was used to determine differences in proportions. The association between the direction of change in W_1_ and the blood pressure was very strong (*P* < 0.0001).

**Table 3:** The contingency table for the chi-square (χ^2^) test

	HT	Norm	Totals
W_1_ inc	24 (72.7%)	14 (26.9%)	38 (44.7%)
W_1_ dec	10 (27.3%)	37 (73.1%)	47 (55.3%)
Totals	34	51	85

The top row (W_1_ inc) shows the number of subjects in whom W_1_ increased during CPT. The second row (W_1_dec) shows the number of subjects in whom W_1_ decreased during CPT. The left column shows the number of hypertensive subjects. The right column shows the number of normotensive subjects

## Discussion

Hypertensive persons are considered to be hyper-reactive to the CPT, and hyper-reactivity to the CPT in normotensive persons is hypothesized to be a marker for future hypertension [1, 2]. However, the mechanism of future development of hypertension in subjects who show an excessive response to the CPT is not established. The current study was not designed to test this hypothesis, but rather aimed to define ‘cardiovascular reactivity’ without ambiguity. A widely accepted indicator for reactivity is the amount of increase in blood pressure. However, the threshold value to discriminate between hyper-reactive and normo-reactive subjects varies with the investigator. The difference in the changes in the maximum carotid arterial blood velocity between HT and Norm, i.e., no change in HT and decrease in Norm, can be a discriminator. The decrease in the maximum velocity is partly explained as follows: carotid arterial stroke volume did not change during CPT, while systolic carotid arterial diameter increased significantly, which caused a reduction in blood velocity. However, systolic carotid arterial diameter increased in HT, as well. Therefore, this reasoning is inconsistent.

We consider that an index comprised of multiple variables would be more informative than blood pressure alone or velocity alone. We evaluated WI, which is defined in terms of both blood pressure and velocity. Changes in WI during CPT were two directional, i.e., increase or decrease. The association between the direction of change in W_1_ (increase or decrease) and blood pressure (HT or Norm) was very strong (*P* < 0.0001) (Table 3). The maximum WI during a cardiac cycle (W_1_) strongly correlates with left ventricular Max dP/dt, an index of cardiac contractility [3, 5–7]. Therefore, in other words, cardiac contractility increases during CPT in HT, but decreases in Norm.

Scatter diagrams for the relationship between %W_1_ and age show that there is a tendency for higher age (say age > 60) to be associated with positive values of %W_1_ both in Fig. 2 left and right. Actually, the difference between the mean values of %W_1_ in the HT and Norm groups is not significant for age > 60. However, in the figure for the Norm group, the slope of the regression line is significantly positive (*r* = 0.58, *P* < 0.0001), while in the figure for the HT group, the slope of the regression line is negative but does not deviate significantly from zero (*r* = –0.19, *P* = 0.30). Thus, %W1 has a tendency to be negative for lower age in the Norm group, while it has no significant association with age in the HT group.

**Fig. 4:**
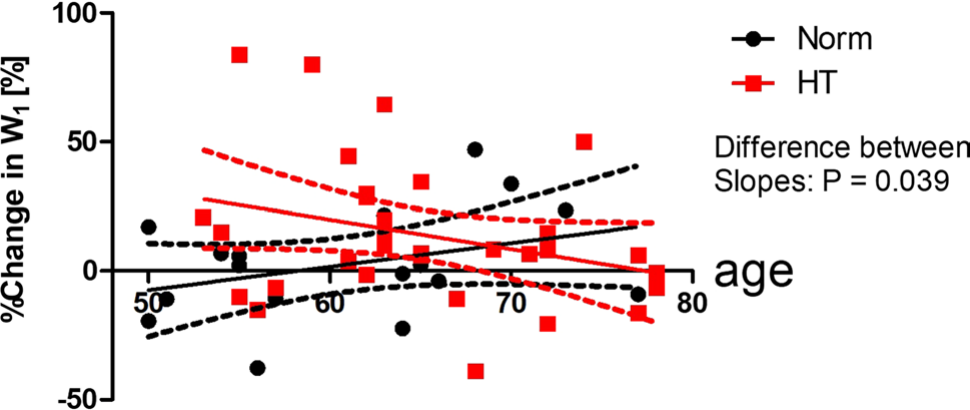
Comparison of the regression lines of the percent changes in W_1_ during CPT on age for the normotensive group and for the hypertensive group over the age range of 50 to 80. Percent changes are normalized to W_1_ before CPT

However, the age ranges of the Norm group and HT group are different (20–80 vs 50–80). To examine whether the above-mentioned tendencies are also observed over the common age range of 50–80, we compared the regression line of %W_1_ on age for the Norm group and that for the HT group in this age range (Fig. 4). The regression line for the Norm group does not deviate significantly from zero (*r* = 0.354, *P* = 0.164). That for the HT group also does not deviate from zero (*r* = – 0.304, *P* = 0.091). Therefore, as far as the age range of 50–80 is concerned, the associations between % W_1_ and age are not significant in either the Norm or HT group.

We also evaluated the effects of stiffness parameter β of the carotid artery before CPT on %W_1_ during CPT (Fig. 3). In Norm, the amount of %W_1_ during CPT is not associated with β. In HT, however, the amount of %W_1_ is associated with β. The effects of the above confounding factors on changes in W_1_ are not consistent. Thus, the mechanisms underlying the two-way changes in cardiac contractility during CPT are largely unknown. Nevertheless, this property of cardiac contractility, i.e., decrease or increase, is a clear marker to discriminate cardiovascular reactivity, which does not vary depending on each investigator’s subjective point of view.

## Limitations

We did not discuss the ability of hyper-reactivity to CPT for predicting future hypertension since we had no data concerning this issue. Our study was limited by the small sample size. Information about smoking status, diabetes mellitus, dyslipidemia, and obesity was not reported, which might have affected the multivariate analyses of factors in relation to the reactions to CPT. Therefore, our findings must be considered to be preliminary until they are replicated by other large studies that are able to show whether there is really no association between these factors and reaction to CPT.

## Conclusion

Wave intensity, an index of ventriculo-arterial interaction, increases in hypertensive subjects, but decreases in normotensive subjects during the cold pressor test. Direction of change in W_1_ during CPT is a marker without ambiguity.
